# Leveraging Technology to Engage Supplemental Nutrition Assistance Program Consumers With Children at Farmers Markets: Qualitative Community-Engaged Approach to App Development

**DOI:** 10.2196/70104

**Published:** 2025-05-16

**Authors:** Callie Ogland-Hand, Jillian Schulte, Owusua Yamoah, Kathryn Poppe, Timothy H Ciesielski, Regan Gee, Ana Claudia Zubieta, Darcy A Freedman

**Affiliations:** 1 Mary Ann Swetland Center for Environmental Health, School of Medicine, Case Western Reserve University, 6700 Euclid Ave, Cleveland, OH, 44103, United States, 1 216 368 3060; 2 Department of Population and Quantitative Health Sciences, School of Medicine, Case Western Reserve University, Cleveland, OH, United States; 3 Department of Anthropology, Case Western Reserve University, Cleveland, OH, United States; 4 College of Food, Agricultural, and Environmental Sciences, The Ohio State University, Columbus, OH, United States

**Keywords:** farmers market, fruit and vegetable consumption, nutrition incentive, SNAP, community-engaged research, mobile app, app development

## Abstract

**Background:**

Fruit and vegetable consumption is lower than national trends among people receiving Supplemental Nutrition Assistance Program (SNAP) benefits due to economic and physical access barriers. Monetary nutrition incentive programs at farmers markets aim to reduce these barriers to improve diet quality among SNAP consumers. We leveraged community-engaged methods to collaboratively design a mobile app to increase the use of both nutrition incentive programs and farmers markets among SNAP households with children. This population represents about 35% of all SNAP households providing the dual benefit of improving diet for both adults and children.

**Objective:**

In this paper, we share the iterative, community-engaged development process used to design a technology intervention that encourages the integration of farmers markets into the food shopping routines of SNAP consumers with children.

**Methods:**

Our qualitative community-engaged approach was informed by human-centered design, following the inspiration and ideation phases of this framework. In the “inspiration” phase, we worked with community nutrition experts to define both the goal of and target audience for the app (ie, SNAP households with children). In the subsequent “ideation” phase, we completed 3 stages of data collection. We developed 2 interface prototypes and received feedback from end users on design and usability preferences before selecting a baseline model. Additional feedback gathered from qualitative interviews with 20 SNAP consumers with children was incorporated into the app’s version 1 (V1) development. We then shared V1 with SNAP consumers, children, and farmers market managers to test the app’s functionality, design, and utility.

**Results:**

In the “inspiration” phase, the community nutrition partners identified SNAP consumers with children younger than 18 years as the target population for the app. In the “ideation” phase, we successfully created V1 through 3 stages of a qualitative, community-engaged process. First, about 75% (n=3) of SNAP consumers and all farmers market managers selected a grocery shopping design option for the layout of the app. Second, we integrated features identified by SNAP consumers with children into the app design, such as market information (ie, location with GPS address links, hours, website), likely available market inventory, market events, and grocery shopping checklists. Finally, we obtained recommendations for future versions of the app, including real-time changes in market hours, additional notification options, and grocery list personalization during a demonstration of V1. Both SNAP consumers and farmers market managers expressed interest in the app’s launch and utility.

**Conclusions:**

It is feasible for community nutrition researchers to successfully design a community-engaged mobile app with the assistance of software developers. The community-engaged approach was key to us integrating potential end users’ preferences in the design of V1. Future work will assess the app’s impact on low-income families’ use of local farmers markets and nutrition incentive programs, as well as fruit and vegetable consumption.

## Introduction

### Background

Most Americans (≈90%) do not meet recommendations for daily fruit and vegetable intake and the trends are even worse for those with low income [1]. People receiving Supplemental Nutrition Assistance Program (SNAP) benefits consume fewer fruits and vegetables compared with people not receiving these benefits [2], and low-income food-insecure households have higher rates of chronic disease [3]. Accordingly, increasing fruit and vegetable consumption is a national priority to reduce chronic disease risk among Americans [1], and this is especially important for low-income households.

Children tend to eat insufficient fruits and vegetables [4-6]. Child fruit and vegetable consumption is impacted by food availability at home, neighborhood food environment, and parents’ perceived produce availability [7]. Relatedly, increased income is directly associated with increased fruit and vegetable consumption, putting children from families with low income at particular risk of not meeting dietary recommendations [4]. Low-income parents with young children represent about 35% of all SNAP households [8]. Financial constraints, limited time, and child food preferences are particularly burdensome for this population [9-12].

Monetary nutrition incentive programs address cost barriers to purchasing fruits and vegetables by augmenting SNAP benefits with additional “incentives” to lower food costs [13-15]. These programs are often available at local farmers markets, and they have successfully improved access to fruits and vegetables, improved fruit and vegetable intake, and reduced food insecurity, particularly for low-income households [16-19]. Despite the benefits of nutrition incentives at farmers markets, lack of awareness about these opportunities limits their use. A recent study in Ohio found only 45% of SNAP consumers with access to nutrition incentive programming within 5 miles of their home were using the program; most of the nonusers (>80%) were unaware that the incentive program existed [20]. Yet, 77% of the nonusers said that they were likely to use the program in the next 6 months after learning about the program [20]. Other research has found lack of awareness is a modifiable barrier to the use of nutrition programs [21,22]. These findings highlight how a lack of awareness of nutrition incentive programs at farmers markets can impede use.

### Design Framework

In line with calls to translate scientific evidence into interventions that increase fruit and vegetable access and consumption, particularly among low-income families [23], our team set out to design a mobile app for SNAP consumers with children. Mobile apps are a popular approach in translational science in behavioral health interventions [24,25], demonstrating some success in changing dietary patterns [26]. Effective dissemination, implementation, and translational science, however, hinges on community engagement, where the target population is engaged in all processes from problem identification to design and implementation of interventions [27]. Community participation in technology development often occurs through the use of “Human-Centered Design”—a collaborative design process that includes multiple stakeholders [28,29]. Human-centered design is a community-engaged framework for technology development [30], and its use can help create buy-in, trust, and use of tools among target populations [31]. Our community-engaged approach was inspired by the iterative process of human-centered design, and our work in this paper will focus on 2 process phases and 3 stages of data collection.

### Goal of This Study

We sought to raise awareness of nutrition incentive programs among SNAP consumers with children by developing a mobile app. To do this we leveraged a qualitative community-engaged process. We chose a community-engaged approach because the technology that is desired and designed by end users should be more useful to them, and it can lead to rapid adoption [30,31]. Ultimately, this approach should identify mobile app features that increase awareness of farmers markets and nutrition incentive programs and provide value to customers and managers of farmers markets.

## Methods

### Overview

**Figure 1.**
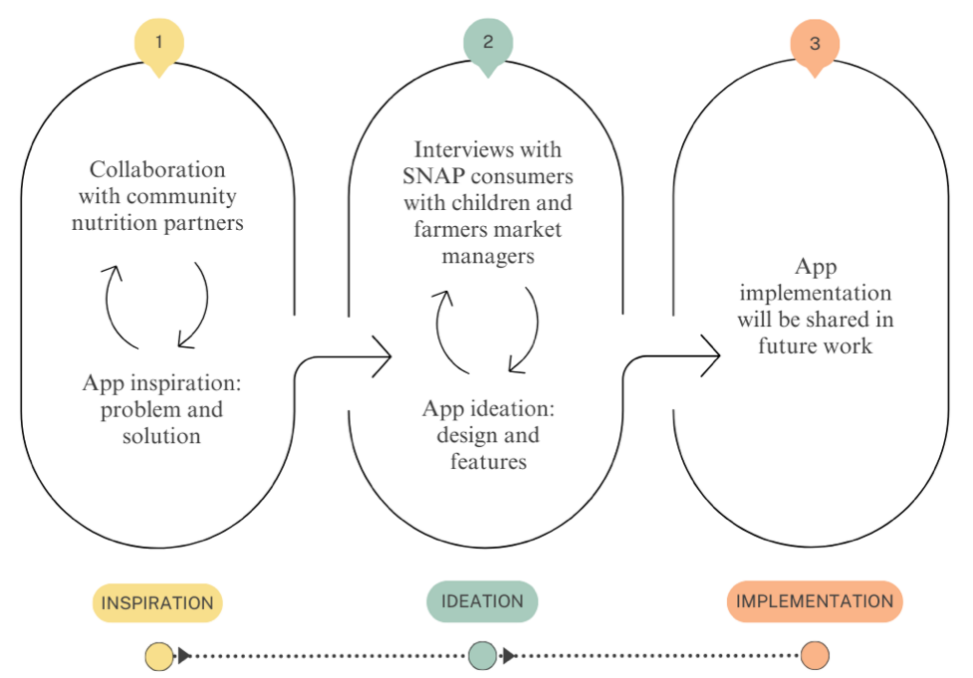
Our development process for the app was an iterative, qualitative, and community-engaged process with 3 phases inspired by human-centered design principles of inspiration, ideation, and implementation [30]. SNAP: Supplemental Nutrition Assistance Program.

The development of the app consisted of three community-engaged phases informed by human-centered design as follows: (1) inspiration, (2) ideation, and (3) implementation [28], outlined in Figure 1. Applying community-engaged concepts to app development has the potential to improve health behaviors, health knowledge, and awareness and use of health benefit programs [32]. Consequently, community-based methods and human-centered design principles have been applied to the field of public health and health care technology tools and services, ranging from physical activity in organ transplant recipients [33], telehealth services [31,34], and the Special Supplemental Nutrition Program for Women, Infant, and Children (WIC) programs [35,36]. Because we wanted to create an app with the aforementioned benefits that would be widely useful, human-centered design was embraced as a guiding framework for this community-engaged work. The inspiration and ideation phases occurred between May 2021 and October 2022, and the implementation phase is still ongoing.

This study was designed and conducted by researchers at Case Western Reserve University in consultation with community nutrition partners at Ohio SNAP-Ed and Produce Perks Midwest, who have expertise in public health, federal food assistance programs (ie, SNAP, WIC), nutrition incentive programs, and farmers markets.

### Phase 1: Inspiration

The “inspiration” phase included collaboration with community nutrition partners to identify the problem and brainstorm possible solutions.

#### Problem Identification

For over a decade, our research team has focused on healthy food access among low-income populations with a focus on farmers markets. Financial costs and physical access barriers limit the consumption of fruit and vegetables, especially among households with low income. While nutrition incentive programs aim to curb financial burdens and encourage farmers market use, awareness of local farmers markets and existing nutrition incentive programs is low among SNAP consumers [20,37-39]. The lack of repeat SNAP consumers at farmers markets is a national issue. Of the SNAP households shopping at a farmers market in 2021, roughly half made only one purchase throughout the year [40]. This underuse may be amplified by additional barriers to farmers market, including location, hours of operation, and transportation [41]. Face-to-face interventions aimed at improving engagement with farmers markets among low-income populations were not widely adopted because of the human capital investment required to support implementation [42], so technology may be an avenue to increase farmers market use.

#### Identifying Solutions

Building on this evidence, researchers from our team along with community nutrition partners tapped into lessons we have learned from our ongoing use of technology to improve community nutrition [22,43,44]. Our goal was to explore technology-based solutions to bridge the awareness gap among SNAP populations related to the availability of nutrition incentive programming.

Our previous work found that the majority of people hear about food procurement opportunities (like farmers markets) and nutrition incentive programs through word-of-mouth [20,45]. We hypothesized that technology could provide a unique opportunity to spur word-of-mouth dissemination [46,47]. Additionally, in support of a technology-based intervention, several studies have found significant improvement in diets and other health behaviors through app use [48-51]. Thus, the idea for a mobile app was identified as a solution to the underuse of farmers markets and nutrition incentive programs among low-income populations.

#### Prior Experience With Digital Solutions for Farmers Markets

Support for a digital technology intervention like the app was strengthened by our research team’s previous successful development and deployment of technology-based solutions to improve community nutrition, including the Produce Path Manager Portal. Produce Path Manager Portal is an app that allows farmers market managers to track transactions, nutrition incentive use, vendors, and general information about their farmers market [52]. In use at over 500 farmers markets across 7 states, Produce Path Manager Portal is designed for market managers and state-level organizations to track and report on nutrition incentive programming. Farmers market managers create individual accounts for their markets, but market information is not accessible publicly for market customers.

#### Proposed Solution: Consumer-Facing Portal

The creation of the new app enhances the market manager features (of Produce Path) as a manager-facing portal and merges those features with a customer-facing portal for market customers. Given their different perspectives and expertise, both managers and consumers were included in our design and development process. We contracted a software development team to help design the prototype for the app and build version 1 (V1). Through a bidding and request-for-proposal process, we invited 2 companies for interviews with our research team and community nutrition partners. We selected one company based on their described skills, previous app development, and ideas for the app.

### Phase 2: Ideation

#### Overview

The “ideation” phase included development and feedback iterations (3 stages) to inform prototype design and learn about interests in using the app as well as features and design preferences. Qualitative feedback was collected over 16 months (May 2021-October 2022) with key end users: SNAP consumers with children and farmers market managers. Data came through feedback sessions and structured web-based interviews conducted via Zoom. Informed consent was obtained from participants involved in structured interviews. A summary of the data collection process and goals is outlined in Table 1.

**Table 1.** Our qualitative, community-engaged process of developing an app, informed by human-centered design: data collection, participants, and goals.

Design phase and goals		Data collection and participants
Phase 1: Inspiration		
	1. Problem identification 2. Identifying solutions	Consultation with community nutrition partners (n=7)
Phase 2: Ideation		
	1. Determining prototype design and app interest	Stage 1 Interviews Feedback sessions with SNAP^a^ consumers (n=4) Feedback sessions with farmers market managers (n=3)
	2. Determining features and design preferences	Stage 2 Interviews Formal interviews with SNAP consumers (n=20)
	3. Feedback on V1	Stage 3 Interviews Feedback sessions with SNAP consumers (n=7) Feedback sessions with farmers market managers (n=6)
Phase 3: Implementation		
	—^b^	—

a SNAP: Supplemental Nutrition Assistance Program.

b Not available (future work).

#### Stage 1 Interviews (November 2021 to February 2022): Determining Prototype Design and App Interest

**Figure 2.**
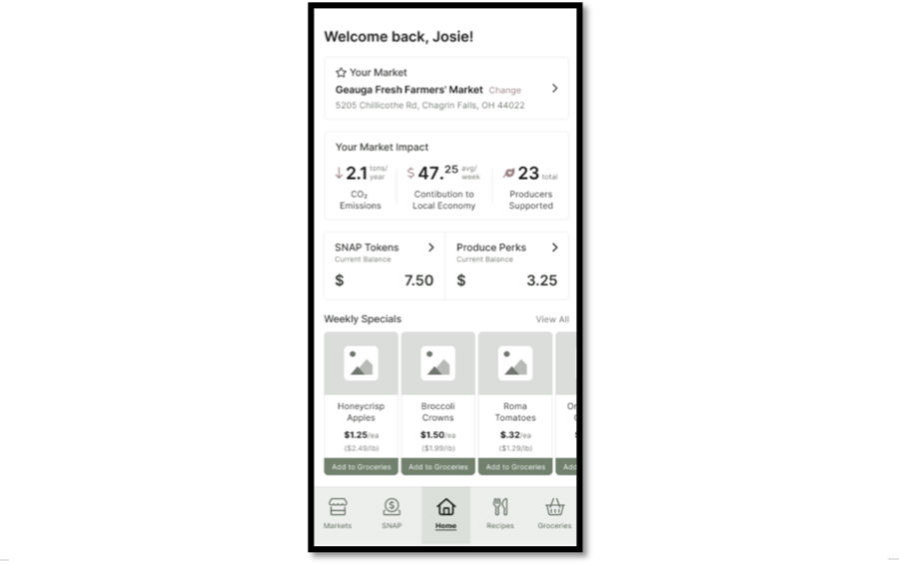
The grocery shopping prototype design, one of 2 app design options designed by our software developers and presented to Supplemental Nutrition Assistance Program (SNAP) consumers for feedback.

Similar to approaches adapted by Kruzan et al [53] and Chandler et al [54] we worked with software developers to create 2 initial prototype interfaces. One was a shopping-centered design with a grocery list option and the second was a game-centered design with rewards and points for using the app. Both prototypes featured recipes with produce from farmers markets, a map of farmers markets, and a digital wallet for managing SNAP benefits, as illustrated in Figures2  3. The shopping-centered design included market inventory and the ability to add produce to a grocery list. The gamification design distributed points to users for using the app and its features, and accumulated points could be traded in for in-app rewards, such as ribbons and badges.

**Figure 3.**
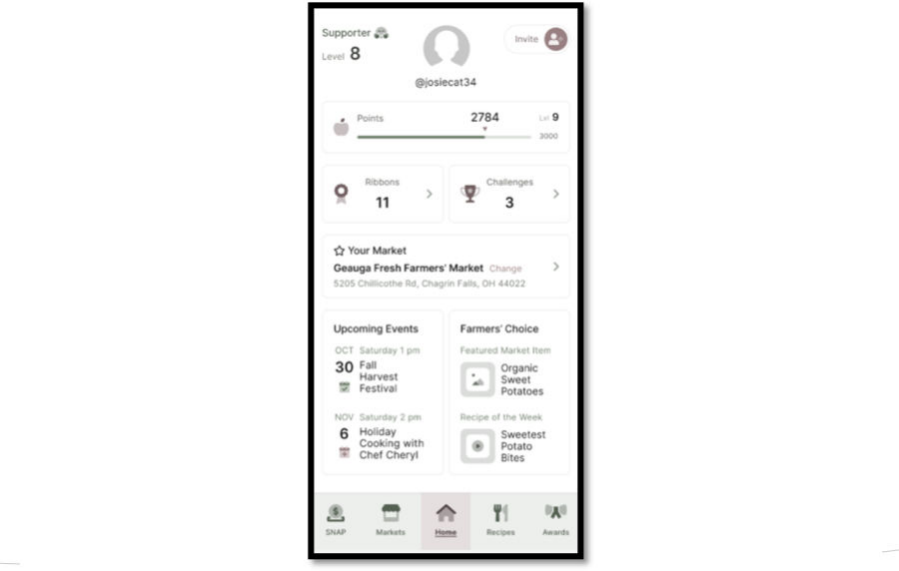
The gamification model prototype design, one of 2 app design options designed by our software developers and presented to Supplemental Nutrition Assistance Program (SNAP) consumers for feedback.

We held web-based feedback sessions via Zoom with SNAP consumers with children who had previous farmers market experience (n=4) and farmers market managers (n=3) to prioritize the prototype designs and understand the desired features of the front-facing app. These participants were purposively recruited by word-of-mouth by our community nutrition partners. During the individual interviews, all participants were presented with simple, low-fidelity versions of the 2 app models and shown the content of the app and how they would function. Participants were asked about their overall impressions of both prototypes (ie, “Were there specific features of this model that you liked? Why?”) and what features would motivate them to keep using the app. Participants were also asked if they thought either of the prototypes could potentially improve their farmers market experiences. Interviews were conducted by researchers COH and KP, and they lasted between 30 and 60 minutes and were not recorded or transcribed.

#### Stage 2 Interviews (July to September 2022): Determining Features and Design Preferences

Following the prototype selection in stage 1, we conducted structured qualitative interviews with 20 SNAP consumers with children younger than 18 years to understand their healthy food shopping routines and features of the app that might align with those routines. These interviews were conducted digitally via Zoom by researchers COH, JS, OY, RG, and KP, and they lasted between 45 and 60 minutes. We collected information about their likes and dislikes of mobile app designs in general, as well as apps that they specifically used in their cooking and shopping routines. These participants were recruited from prior research studies and across the state of Ohio through phone calls and flyers at farmers markets, grocery stores, and program offices (ie, WIC, Head Start). Eligible participants had used SNAP in the last 12 months, had at least 1 child younger than 18 years at home half of the time, did at least half of the household grocery shopping, and had experience using a smartphone app.

The interview guide and our engagement with the potential end users were rooted in the diffusion of innovation (DOI) theory. DOI is one of the first social science theories to understand behavior change [55]. This theory considers both diffusion, meaning the passive spread of an idea or product over time, and innovation, which includes novel ideas, products, interventions, etc [56]. Recently, DOI has been applied to health-related technology and mobile apps to understand their adoption and scalability [57-61]. The interview questions were shaped by 3 DOI attributes [56]: (1) relative advantage (ie, “Can you tell me about any apps or websites that you use for preparing and planning for shopping and cooking for your family?”), (2) compatibility (ie, “How would you rate this statement on a scale of 1, meaning not a good fit, and 10, a perfect fit: Using an app that tells me what foods are available at the farmers market every week could help me add more fresh and healthy foods to my shopping list?”), and (3) complexity (ie, What do you dislike about the apps that you use, or have used in the past?”). Attending to these constructs of innovations can aid in adoption and diffusion [55,62].

Following each interview, the interviewer and notetaker met to debrief and recorded key features of interest and any feedback relevant to the app development on a debrief form. Preliminary rapid analysis of debrief forms and discussions among the researchers who were involved in the interviews revealed features and design ideas mentioned by participants throughout the interviews. Informed by participants, research team members, community partners, and software developers, some suggested features were incorporated into the development of V1 in real time based on feasibility, available resources, and time. All interviews were recorded and transcribed, and formal qualitative analysis will be presented in future work.

#### Stage 3 Interviews (October to November 2022): Feedback on Version 1

Finally, individual feedback sessions were held with SNAP consumers (n=7) and farmers market managers (n=6) regarding V1 of the app. All SNAP consumers in these feedback sessions had previously participated in the stage 2 interviews. Two farmers market managers had previously participated in stage 1 interviews, and the remaining were 4 were purposively recruited by word-of-mouth by our community nutrition partners. SNAP users were selected based on the relevance of their responses in the stage 2 interviews related to app development and app use experience. During the debrief sessions after each interview in stage 2, the interviewer and note taker rated the relevance of participants’ responses to the research goals on a scale of 0 (low) to 10 (high). Participants who scored 8 or higher were invited to participate in stage 3 feedback sessions. In these feedback sessions, V1 was shared to test the functionality, design, and utility of the app.

During these individual interviews, researchers demonstrated the app; SNAP consumers were shown the consumer-facing portal and market managers were shown the manager-facing portal to market managers. After the demonstration, participants were given time to practice using the app on their own (ie, SNAP consumers added certain items to their grocery list and market managers created a new market event). All participants were asked about their likes and dislikes (ie, “How well will the app support farmers market use and experience for you and other families with children who shop with SNAP?”), pains and gains (ie, “What feature(s) was confusing and why? What will help make it less confusing?”), and ideas for additional features (ie, “From your perspective, what kind of information should be included in a marketing video about farmers markets and shopping with SNAP at farmers markets?”). SNAP consumers were asked about the functionality of the shopping list and market selection, whereas market managers were asked about the functionality of updating market inventory, events, and vendors. The goal of these pilot interviews was to inform future app updates.

### Phase 3: Implementation

The final stage of this work, as it is guided by human-centered design concepts, consists of iterations of measuring and learning. The implementation research testing the app will be presented in future work, along with pilot results.

### Ethical Considerations

This study was approved by the Case Western Reserve University Institutional Review Board (STUDY20220359). All participants consented verbally to the study, and no specific quotations or identifiable details have been shared in this paper. SNAP consumers in the stage 2 (formal semistructured qualitative) interviews were compensated with a US$ 30 e-gift card to a superstore for their time.

## Results

### Phase 1: Inspiration (Problem Identification)

To improve awareness of and access to farmers markets among SNAP consumers, we needed to answer the following questions: (1) how is information about farmers markets and nutrition incentives shared with SNAP consumers? (2) What information do SNAP consumers need and want? Collaboration with our community partners led us to further focus this research question on a specific population—low-income parents—who face additional challenges and barriers to healthy food shopping and engagement with local farmers markets [9-12].

### Phase 2: Ideation

#### Stage 1 Interviews: Determining Prototype Design and App Interest

Feedback sessions in stage 1 determined the prototype design. Core features of both prototypes included: market home pages with the address, hours, and website in addition to inventory, vendors, and events tabs; a map of nearby markets with the ability to “star” a home market; a recipe bank that could be filtered by ingredient; and, a web-based SNAP wallet that tracked both EBT and nutrition incentive use. The grocery shopping model uniquely included a customizable grocery list and the recipe ingredients could be directly added to the grocery list. On the other hand, the gamification version included an awards page to encourage app use, including challenges to visit multiple farmers markets and make recipes with leftovers, and in-app engagement to favorite recipes and review markets.

About 75% (n=3) of the SNAP consumers interviewed were highly in favor of a grocery model design compared with the gamification model (Figure 2 can be used for comparison). Their choice was largely informed by the utility of a grocery list, and not feeling motivated by in-app rewards or points in the game version. One participant for example shared that earning badges in the gamification version would not motivate them to use the app since they could not use those rewards in “real life.” Another shared that they felt farmers market users would like the grocery shopping model since it provides exactly what they need. Thus, we moved forward with the grocery model for the app development. Participants expressed general interest in the app, stating that app-available information could help them eat healthier, and they emphasized the potential for the app to save them time and money while grocery shopping.

All farmers market managers were similarly interested in the app and were shown the customer-facing portal. They particularly liked the feature that listed the available produce at their markets, along with the option to indicate if a product was limited in quantity at their market. They felt the app would help them engage with market customers, and that it would help customers prepare for visits to the market.

There was mixed feedback on the web-based SNAP wallet feature and the feasibility of linking EBT cards through the app: some SNAP consumers expressed distrust in digital finances, and some market managers were concerned about market vendors not having the technology to accept digital payments. Thus, we decided to not pursue this feature in V1 development. Additionally, participants expressed a desire for a clean, non-cluttered design. Participants were distracted by having too much information on the home screen.

#### Stage 2 Interviews: Determining Features and Design Preferences

**Figure 4.**
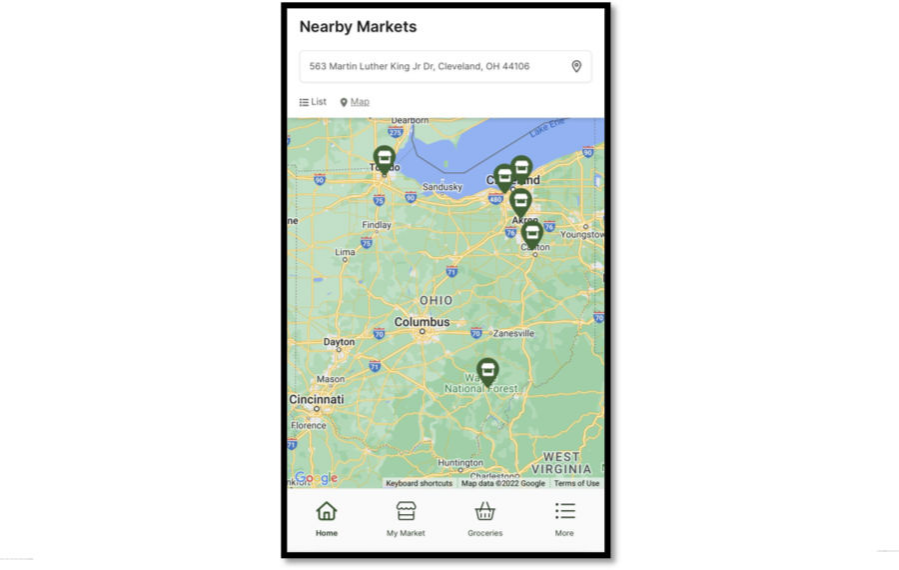
Example features that were incorporated from human-centered design phase 2, stage 2 interviews with Supplemental Nutrition Assistance Program (SNAP) consumers: a map of markets based on location or inputted address.

**Figure 5.**
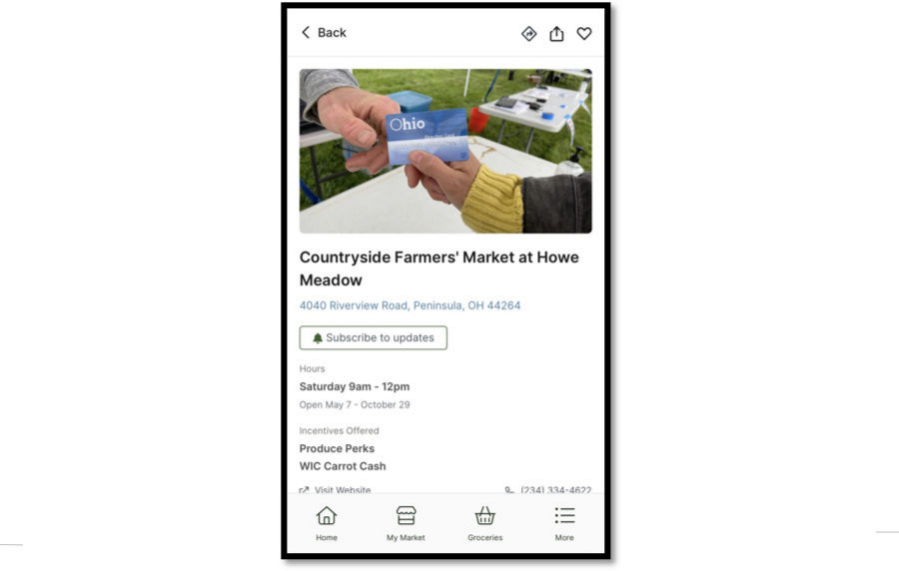
Example features that were incorporated from human-centered design phase 2, stage 2 interviews with Supplemental Nutrition Assistance Program (SNAP) consumers: hyperlinked market address that links to a navigation app.

**Figure 6.**
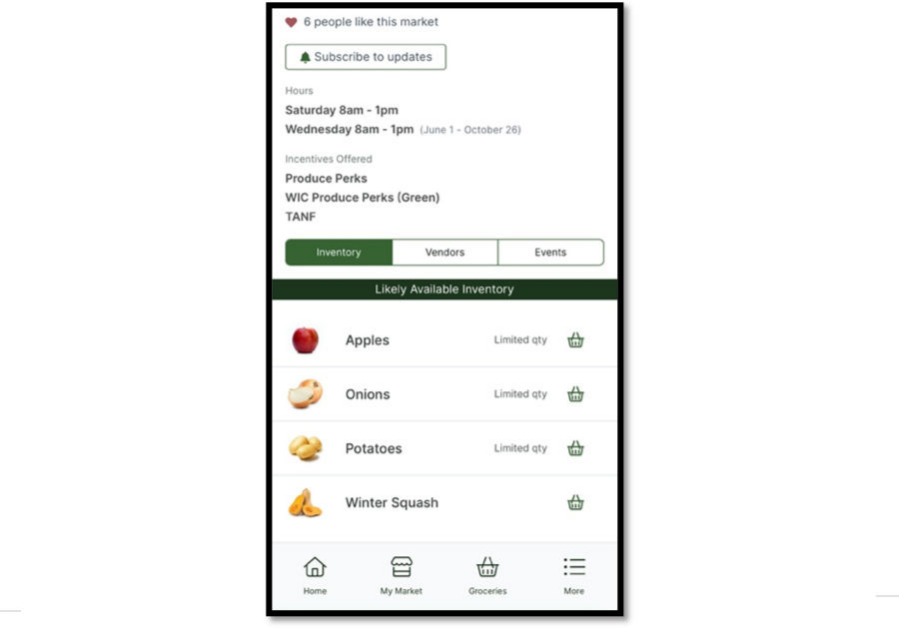
Example features that were incorporated from human-centered design phase 2, stage 2 interviews with Supplemental Nutrition Assistance Program (SNAP) consumers: likely available inventory at a specific market with the option to add to groceries by clicking on the basket icon.

Formal qualitative interviews with SNAP consumers in stage 2 provided feedback on the desired features and design of the app, as well as overall utility. Participants were mostly Black (n=10, 50%), women (n=19, 95%) with children between 0 and 5 years, and lived in urban counties (n=14, 70%) in Ohio (Table 2 contains demographic characteristics of participants). About 80% (n=16) of the participants used mobile apps daily in their routine. Many suggested features and functions from stage 2 interviews were implemented in real-time development based on participant consensus and feasibility from our software design partners, as illustrated in Figures 4-6. Cost and time prevented us from incorporating all suggested features, such as features to manage nutrition incentive balances.

**Table 2.** Characteristics of stage 2 interview participants (Supplemental Nutrition Assistance Program [SNAP] consumers, n=20).

	Participants (n=20)
Gender, n (%)	
Female	19 (95)
Male	1 (5)
Age (years), mean (range)	37.8 (24-54)
Race and ethnicity, n (%)	
Black	10 (50)
White	6 (30)
Latinx	2 (10)
Unidentified	2 (20)
Parent or caretaker of children aged 0‐5 years, n (%)	14 (70)
Number of children aged 0‐5 years in these households, mean (range)	1.8 (1-4)
Parent or caretaker of children aged 6‐11 years, n (%)	13 (65)
Number of children aged 6‐11 years in these households, mean (range)	1.5 (1-4)
Parent or caretaker of children aged 12‐17 years, n (%)	7 (35)
Number of children aged 12‐17 years in these households, mean (range)	1.3 (1-3)
County of residence^a^ n (%)	
Urban counties	14 (70)
Rural counties	6 (30)
Employment status, n (%)	
Employed full-time	4 (20)
Employed part-time	6 (30)
Currently out of work	3 (15)
Homemaker	6 (30)
Unable to work	1 (5)
2021 total household income (US $), n (%)	
Less than $25,000	13 (65)
Between $25,000 and $34,999	3 (15)
Between $35,000 and $49,999	3 (15)
Between $50,000 and $74,999	1 (5)
In a typical month, how involved are you in food shopping for your household?, n (%)	
I do more than half of the shopping	1 (5)
I do all the shopping	19 (95)
Have you ever used Produce Perks^b^?, n (%)	
Yes	9 (45)
No	10 (50)
I don’t know	1 (5)
In a typical month, how often do you use an app on your smartphone?, n (%)	
Never	1 (5)
Weekly	3 (15)
Daily	16 (80)

a Urban and rural county designations are based on the Ohio Department of Health classification. Rural and partially rural counties are classified as rural in this analysis.

b Produce Perks is the SNAP-based fruit and vegetable incentive program in Ohio.

Consumer suggestions included in V1:

- List of likely available inventory.
- List of farmers market vendors.
- Highlight markets that accept incentive programs.
- Map of nearby farmers markets based on user location.
- Hyperlink market address to external GPS app.
- Share farmers market events with the ability to add them to the external calendar app.
- Include recipes with produce from the market.
- Grocery list function with the ability to cross items off.
- Instructions on how to use nutrition incentives.
- Up-to-date content.

#### Stage 3 Interviews: Feedback on Version 1

After incorporating the previously mentioned suggestions from stage 2 interviews, we finalized the app V1 development and informally shared it with 7 SNAP consumers from the stage 2 interviews and 6 farmers market managers representing markets in rural, urban, and diverse racial populations. The SNAP consumers were all women, 43% (n=3) identified as Black and another 43% (n=3) identified as White, about 14% (n=1) were Hispanic, and 57% (n=4) were from urban counties in Ohio. SNAP consumers thought the app was clear and straightforward. They provided additional features and design suggestions to inform the app version 2 and future app updates, including:

- Add links to vendors’ websites or social media handles.
- List nonproduce inventory at the market, such as baked goods, craft items, and dairy products; then, add filter options by item category.
- Add custom items to the grocery list, in addition to the inventory items at the market, and the ability to undo crossing an item off the list.
- Rethink the home screen to include users’ preferred farmers market.
- App branding incorporated throughout the app.

Farmers market managers found the manager-facing portal of the app V1 simple, user-friendly, easy to navigate, and visually appealing. The manager-facing portal was created from Produce Path Manager Portal, which they were familiar with, and offered enhanced features. Market managers appreciated the messaging feature and discussed that it could replace some market newsletters and offer more streamlined communication between markets and customers. Farmers market managers expressed the following suggested updates:

- Increase customization features to add more information about vendors, inventory, and general market details.
- Add analytics on customers who use the app, such as what items customers are adding to their grocery list to track product demand.
- Ability to add real-time open and closed dates and times for their market.

## Discussion

### Principal Findings

By using a qualitative community-engaged approach to app development, SNAP consumers and farmers market managers were involved throughout the design phases of the app to raise awareness about nutrition incentive programs and farmers markets in general. We found SNAP consumers and farmers market managers were interested in the grocery shopping model compared with the gamified version of the app. Through the interactive feedback and design process, participants suggested key features for the app including listing likely available inventory including nonproduce items, mapping nearby markets based on location, providing information about nutrition incentive programs, and connecting market addresses and events to outside apps (maps and calendars).

Centering partnerships between researchers, community members, stakeholders, and community partners are essential to creating equitable research [63]. The collaborative nature of community-engaged research allows community members and the target end users to make key design decisions and ensure that the developed technology meets their needs and desires. In this way, community engagement is a recommended method to work toward equity [29,64]. It is important to note that translational research requires the diverse partnerships that community-engagement and human-centered design promote. Translation, or the process of the widespread use of evidence-based programs, practices, and policies, has been a long-time priority of the Centers for Disease and Control to help prevent chronic disease [65]. Successful translation of products, such as a mobile app, necessitates input from diverse participants involved in product development [65]. Our research is strengthened by the collaborations we forged with OSU SNAP-Ed, Produce Perks Midwest, farmers markets throughout Ohio, SNAP consumers, and our contracted software developers.

Researchers aiming to develop mobile apps through similar processes should be aware that the collaborative and iterative nature of community-engaged methods is time- and resource-intensive. Data collection to inform app design and development occurred over the course of 16 months. For the app to remain useful and functioning well, consistent maintenance and a long-term contract with our app developers are essential. Indeed, work to improve and update the app is ongoing to date, and there are future plans for planning and piloting studies to test efficacy, as well as implementation research to assess app adoption.

This research aligns with growing interest in community-based approaches and human-centered design within public health [66], and the prior research on app development specifically for WIC participants [35,36,67-69]. This research illuminated that WIC participants prefer an app that allows them to locate nearby WIC offices, scan WIC foods at stores, provide appointment reminders, generate shopping lists, share recipe galleries, and can be navigated with minimal difficulty [35,67,68].

There is growing research on the need for more “human centric mobile apps” for federal assistant programs, such as SNAP [70,71]. The Ohio Department of Job and Family Services launched the mobile app ConnectEBT, but this primarily functions as a digital wallet and does not include other features relevant to food shopping for EBT users [72]. This is a significant gap in research, as one study found that WIC participants using a WIC-related app had higher rates of full redemption, thus maximizing their food benefits [73]. Together, these prior studies demonstrate the potential for technology to encourage healthy eating behaviors among low-income populations. These prior findings motivate our approach and support the use of end user input in mobile app development.

### Limitations

One primary limitation of our approach is the number of SNAP consumers (n=24 unique participants) and market managers (n=7 unique participants) that were involved, and this might impact the accuracy of our development. It is also possible that these individuals do not represent consumers and managers outside of Ohio, and this will affect our precision. Thus, we do not claim that this app is optimally tuned for all SNAP consumers or farmers market managers, but these issues can be addressed in future work. Some regional or demographic tailoring may be needed if the app is applied in other settings, as well as language translation if someone has limited English proficiency. Another major limitation of all technology research, including our developed app, is that it requires a level of digital literacy, which may render it inaccessible for some, given the national digital divide [74]. The SNAP consumers and farmers market managers involved in our research had varying levels of comfort using technology and familiarity with apps in general. A new focus on these “digital determinants of health” is increasingly important as health apps become more widely available [75]. Additionally, our research team did not have software design expertise, so the physical development of the app was contracted outside of our institution. This process requires financial and time investments, and the nature of app development requires long-term maintenance. Finally, this app was designed to improve awareness of farmers markets and nutrition incentive programs to help low-income families integrate more fruits and vegetables into their food shopping, but the app features cannot alleviate all access barriers, such as transportation to/from the markets and hours of operation.

### Conclusions

Community-engaged methods, informed by human-centered design principles, are both a viable and uniquely beneficial approach to apply to health intervention app development. Both SNAP consumers and farmers market managers were excited by the prospect of the app and expressed desire for its launch and utility in its functions, highlighting the importance of engaging potential end users in technology development and centering their needs. Our goal is that the continued involvement of SNAP consumers and farmers market managers in the development process will promote buy-in and encourage the iterative development of an equitable, accessible app. Pilot studies of the app V1 and development of version 2 will be presented in future work.

## Abbreviations

DOI: diffusion of innovation
SNAP: Supplemental Nutrition Assistance Program
V1: version 1
WIC: Women, Infants, and Children
